# Development of Polyvinyl Alcohol (PVA) Nanofibers Containing Cationic Lipid/siRNA Complexes via Electrospinning: The Impact of PVA Characterization

**DOI:** 10.3390/nano14131083

**Published:** 2024-06-24

**Authors:** Miyu Kanamori, Kouji Hara, Eriko Yamazoe, Takaaki Ito, Kohei Tahara

**Affiliations:** 1 Laboratory of Pharmaceutical Engineering, Gifu Pharmaceutical University, 1-25-4 Daigaku-nishi, Gifu 501-1196, Japanyamazoe-e@gifu-pu.ac.jp (E.Y.); ito-ta@gifu-pu.ac.jp (T.I.); 2 Laboratory of Nanofiber Technology, Gifu Pharmaceutical University, 1-25-4 Daigaku-nishi, Gifu 501-1196, Japan; hara-ko@gifu-pu.ac.jp; 3 Global Food/Healthcare Department, Mitsubishi Chemical Corporation, 1-1-1 Marunouchi, Chiyoda, Tokyo 100-8251, Japan

**Keywords:** polyvinyl alcohol, nanofibers, electrospinning, siRNA, cationic lipid

## Abstract

This study aimed to develop polyvinyl alcohol (PVA) nanofibers encapsulating 1,2-dioleoyl-3-trimethylammonium-propane (DOTAP)/siRNA complexes via electrospinning for the delivery of nucleic acid-based drugs. It also focused on the influence of the intrinsic properties of PVA on the efficacy of the system. PVA nanofibers, with diameters of 300–400 nm, were obtained, within which the siRNA remained intact and the DOTAP/siRNA complexes were uniformly dispersed. By incorporating DOTAP/siRNA complexes into the PVA nanofibers and assessing the impact of their RNA interference (RNAi) activity in A549-Luc cells, a stable inhibition of luciferase expression was observed. An examination of the nanofiber preparation process revealed that even when DOTAP or siRNA were added separately to the PVA solution without forming complexes, the RNAi effect was retained. The DOTAP/siRNA complexes released from the PVA nanofibers were internalized by the cells, with some PVA residues remaining on their surfaces. The significance of the degree of hydrolysis and polymerization of PVA on the performance of nanofibers was highlighted. Notably, PVA with a low degree of hydrolysis substantially enhanced RNAi effects, with luciferase expression inhibition reaching 91.5 ± 0.7%. Nanofibers made of PVA grades with anionic or cationic modifications were also evaluated, suggesting that they affect the efficacy of siRNA delivery. The insights obtained suggest avenues for future research to optimize drug delivery systems further.

## 1. Introduction

Electrospinning is a technique employed for fabricating nanofibers by applying high-voltage electrostatic forces to a polymer solution within a needle-like nozzle. The physical properties of the nanofibers can be adjusted by altering the polymer, solvent, and parameters such as solution flow rate, as well as applied voltage. Electrospinning is notable for its rapid solvent evaporation owing to the high surface area of the nanofibers, which enables manufacturing at room temperature. This ability makes it a promising technique for applications in tissue engineering, regenerative medicine, preparing wound dressing materials, and synthesizing pharmaceutical formulations [1]. Generally, electrospinning involves applying high voltages ranging from 10 to 40 kV to the solution, but with a very low current in the microampere range; thus, energy consumption is also low. This process, conducted at room temperature, is suitable for unstable biomolecules and advantageous for nucleic acid-based pharmaceuticals despite challenges in productivity [2].

Incorporating nucleic acids, such as siRNA and mRNA, into nanofiber scaffolds to imitate the extracellular matrix is crucial for tissue and stem cell engineering [3,4,5,6,7]. siRNAs encapsulated within nanofiber scaffolds function as controlled-release reservoirs, providing potentially long-term gene therapeutic effects as well as guiding and supporting seeded cells [8,9]. For this purpose, hydrophobic polymers, which are less soluble in cell culture media, such as polycaprolactone or poly(lactide-co-glycolide), are often used as matrices [10].

Polymer nanofibers produced by electrospinning are suitable for transdermal treatment systems and fabricating wound dressing materials [11,12]. Moreover, there have been numerous reports on their applicability in site-specific drug release in the gastrointestinal tract via oral administration, mucosal vaccines for sublingual application, and localized drug therapy in mucosal tissues such as the eyes, lungs, and vagina [13,14,15,16,17,18]. The development of nucleic acid pharmaceuticals incorporated into these polymer matrix nanofibers is anticipated. Electrospinning, as a room-temperature process, is a promising alternative to freeze-drying for solidifying nucleic acid pharmaceuticals. In cases where nanofibers are directly applied to patients, polymers with high water solubility that eventually dissolve or degrade are more likely to be selected.

This study used water-soluble polyvinyl alcohol (PVA), a prevalent excipient in pharmaceutical formulations, as a foundational matrix for nanofiber-encapsulating siRNAs, a category of nucleic acid therapeutics. Recent reports have demonstrated the virtue of PVA nanofibers in drug delivery applications, highlighting their potential for controlled release and biocompatibility [19,20,21].

The physical characteristics of PVA, which predominantly include its water solubility, are determined by its degree of hydrolysis (ratio of hydroxyl to acetyl groups) and polymerization (number of monomers) [22]. The selection of PVA grades allows for tailored water affinity, rendering PVA nanofibers potent candidates for the controlled release of nucleic acid drugs. Through cross-linking, PVA can be rendered insoluble, a property leveraged in preparing wound dressing materials. Additionally, materials that are not inherently suitable for electrospinning into nanofibers, such as carboxymethyl cellulose, can be engineered into nanofibers via hybridization with PVA [23]. Numerous applications for hybrid nanofibers that combine PVA with various functional polymers have been documented [24]. For instance, gellan/PVA nanofibers exhibiting mucosal adhesion and gastric retention [25], gelatin/PVA nanofibers optimized for wound healing [26], and chitosan/PVA and polyvinylpyrrolidone/PVA nanofibers for transdermal drug delivery systems have been developed [27,28]. Consequently, a comprehensive analysis focusing on the encapsulation efficiency of nucleic acid drugs and their cellular uptake efficiency in relation to the different grades of PVA is imperative to optimally harness PVA-based nanofibers as a versatile platform for delivering nucleic acid drug formulation.

In nanofiber-based formulations of nucleic acid drugs, the integration of cationic lipids or alternative transfection agents is essential. Typically, positively charged complexes formed with nucleic acid drugs engage in electrostatic interactions with negatively charged cell membranes, facilitating their adsorption. Subsequently, these complexes are internalized by the cells via endocytosis, which is critical for the functioning of the drugs.

This study aimed to fabricate siRNA-laden nanofibers by electrospinning, and siRNA was employed as a nucleic acid-based therapeutic agent. For siRNA transfection, 1,2-dioleoyl-3-trimethylammonium-propane (DOTAP), a cationic lipid with a quaternary ammonium group, was chosen [29]. Our approach involved embedding the DOTAP/siRNA complex within PVA nanofibers through electrospinning. PVA with a hydrolysis degree of 88% and high polymerization (GOHSENOL™ EG-40P), commonly used as a pharmaceutical excipient, served as the standard. We first prepared DOTAP/siRNA-encapsulated PVA nanofibers using EG-40P to optimize the preparation methods. The different preparation methodologies on the intracellular distribution and RNA interference (RNAi) efficacy of the siRNA was assessed using EG-40P nanofibers. This evaluation was conducted on A549-Luc cells, which are derived from human non-small cell lung cancer and exhibit stable luciferase expression. Additionally, the influence of varying PVA grades, distinguished by their degree of polymerization and saponification, on the inhibition of luciferase activity by siRNA was explored.

## 2. Materials and Methods

### 2.1. Materials

PVAs (GOHSENOL™ EG-40P, EG-05P, EG-30P, KH-20, NH-18, GOHSENX™ K-434, and T-330) were provided by the Mitsubishi Chemical Corporation (Tokyo, Japan). Table 1 presents the degrees of polymerization and hydrolysis of PVAs used in this study. PVAs are abbreviated according to their degrees of polymerization and hydrolysis; for example, PVA24-88 has a 24 × 10^2^ degree of polymerization and an 88% degree of hydrolysis. Annealed siRNA targeting pGL3 firefly luciferase (Luc-siRNA, sense: 5′-CUUACGCUGAGUACUUCGAdTdT-3′, and antisense: 3′-dTdTGAAUGCGACUCAUGAAGCU-5′) was purchased from Nippon Gene (Tokyo, Japan). DOTAP (cationic lipid) was obtained from Avanti Polar Lipids (Alabaster, AL, USA). The Hoechst 33342 stain was purchased from Fujifilm Wako Pure Chemical Corporation (Osaka, Japan). All other chemicals used were of the highest commercially available grade.

### 2.2. Preparation of DOTAP/siRNA Complexes

To prepare DOTAP/sRNA complexes with a nitrogen-to-phosphorus (N–P) ratio of 5, 10, or 100 μL of 20 μM siRNA solution in Milli-Q water was added to an equal volume of DOTAP suspension in Milli-Q water (the final volume was 200 μL, and the final concentration of siRNA was 10 μM).

### 2.3. Preparation of siRNA-Loaded PVA Nanofibers by Electrospinning

In Method A, 25 g of 8% or 20% (*w/w*) PVA aqueous solution was prepared, to which 200 μL of either siRNA solution or DOTAP/siRNA complex (10 μM siRNA) was added. As an alternative approach, in Method B, DOTAP or siRNA were added separately to the PVA aqueous solution to prepare a precursor for electrospinning. To incorporate fluorescein isothiocyanate-labeled PVA (FITC-PVA), prepared as per a previously reported method [30], into the nanofibers, a PVA solution with a ratio of unlabeled PVA to FITC-PVA at 1:9 (*w/w*) was employed. This mixture was then loaded into a 1 mL syringe connected via silicone tubing to a 22G non-beveled needle with an inner diameter of 0.4 mm (Terumo, Tokyo, Japan). Electrospinning of the nanofibers onto a plate-type collector was facilitated by a high voltage of 10 kV from an HVU-30P100 high-voltage power supply (MECC Co., Ltd., Fukuoka, Japan) [22]. The distance between the needle and the collector was maintained at 12 cm, and the solution was delivered to the needle at a flow rate of 0.5 mL/h using a syringe pump (Yutaka Electronics Manufacturing, Gifu, Japan).

### 2.4. Physicochemical Properties of the siRNA-Loaded PVA Nanofibers

The particle size and zeta potential of the DOTAP/siRNA complexes, immediately after preparation and after release from the PVA nanofibers dissolved in Milli-Q water, were measured after appropriate dilution by employing a Zetasizer Nano ZS instrument (Malvern, Worcestershire, UK). The nanofiber mats prepared by electrospinning were imaged using a JSM-6510LV scanning electron microscope (SEM; JEOL, Tokyo, Japan). The mean and standard deviation of the fiber diameter were calculated by measuring 100 randomly selected points on SEM images using the ImageJ™ image analysis software, version 1.53, created by Wayne Rasband, National Institutes of Health, Bethesda, MD, USA, accessed on 20 May 2022, from https://imagej.net/ij/. The siRNA content of the recovered nanofibers dissolved in Milli-Q water was determined using high-performance liquid chromatography (HPLC). For siRNA detection, an EXTREMA HPLC System (JASCO, Tokyo, Japan) equipped with a Waters Xselect CSH C18 column (5 μm, 250 × 4.6 mm i.d.) was used. The detection wavelength was 260 nm. The mobile phase consisted of 0.1 M ammonium bicarbonate solution + acetonitrile in a ratio of 80:20 (*v/v*). The analysis was performed using an isocratic method with a sample injection volume of 10 μL, a solvent flow rate of 1 mL/min, and a column temperature of 30℃. The encapsulation efficiency (%) was ascertained by dividing the amount of siRNA detected using HPLC by the initial amount of siRNA added during the preparation of the nanofibers. PVA nanofibers loaded with FITC-PVA and carboxy tetramethyl rhodamine (TAMRA)-labeled siRNA were observed using an LSM-700 confocal laser scanning microscopy (CLSM; Carl Zeiss, Oberkochen, Germany).

### 2.5. Cell Line and Culture

The A549-Luc cell line (#JCRB1414) was obtained from the JCRB Cell Bank (National Institute of Biomedical Innovation, Osaka, Japan). It was maintained in E-MEM (051-07615; Fujifilm Wako Pure Chemical) supplemented with 10% (*v/v*) fetal bovine serum (Lot No. 1638593; Sigma, Tokyo, Japan), 1% MEM non-essential amino acids (139-15651; Fujifilm Wako Pure Chemical), and 1% penicillin-streptomycin (15140122; Thermo Fisher Scientific, Waltham, MA, USA) at 37℃ under 5% CO_2_.,

### 2.6. Evaluation of the Luciferase-Knockdown Efficiency of siRNA in A549-Luc Cells

A549-Luc cells were seeded in a 24-well plate at a cell density of 2.5 × 10^4^ cells/cm^2^ and cultured for 24 h before treatment. A suspension of the DOTAP/siRNA complexes diluted in a serum-free medium was used for this evaluation. Additionally, all types of siRNA-containing PVA nanofibers were fully dissolved in the serum-free medium, releasing the DOTAP/siRNA complexes, and they were employed for the examination. In this experiment, the PVA nanofibers were dissolved for >30 min, and the absence of any PVA nanofiber remnants in the solution was confirmed before adding it to the cells. After washing the cells once with Hanks’ balanced salt solution (HBSS), 0.5 mL of each sample was added to each well to achieve a final siRNA concentration of 100 nM. Then, they were incubated for 4 h in a CO_2_ incubator. Next, the samples were removed, and the cells were washed with HBSS. Subsequently, the culture medium was added, and the cells were further cultured for 22 h in the CO_2_ incubator. After this, the cells were lysed with a Reporter Lysis 5X Buffer (Promega, Madison, WI, USA) and collected by centrifuging the cell lysates at 18,000× *g* for 2 min and 4 °C. The luciferase activity in the collected supernatant was quantified by employing the PicaGene Luminescence Kit (Toyo Ink, Tokyo, Japan), followed by measuring the luminescence with a GloMax^®^ 20/20 Luminometer (Promega, Tokyo, Japan). The protein concentration was determined using the BCA Protein Assay (Pierce, Rockford, IL, USA), and the luciferase activity was converted to relative light units/mg of protein.

### 2.7. Intracellular Distribution of the siRNA in A549-Luc Cells Observed by CLSM

A549-Luc cells were seeded onto the Lab-Tek^®^ II Chambered #1.5 German Coverglass System (Nalge Nunc International, Naperville, IL, USA) at a density of 2.5 × 10^4^ cells/cm^2^ and incubated for 24 h. Subsequently, the cells were washed with HBSS and treated with DOTAP/TAMRA-siRNA complexes (N/P ratio = 5) encapsulated within FITC-PVA (EG-40P) nanofibers, which were fully dissolved in a serum-free medium to a final siRNA concentration of 100 nM. Of this, 500 µL was added to each well. After incubation for 4 h in a CO_2_ incubator, the siRNA samples were removed, and the cells were washed with HBSS. Cell nuclei were stained by incubation with 10 μg/mL Hoechst 33342 for 20 min. After washing with HBSS, the cells were fixed with 200 µL of 4% paraformaldehyde. The intracellular fluorescence distribution was ascertained using an LSM-700 CLSM.

### 2.8. Cytotoxicity Assays

A549-Luc cells were seeded in a 96-well plate at a density of 7.81 × 10^4^ cells/cm^2^ and incubated for 24 h. The cells were then washed with HBSS and treated with DOTAP/siRNA (N/P ratio = 5)-containing PVA nanofibers fully dissolved in a serum-free medium. The solution was adjusted to a final siRNA concentration of 100 nM by adding 0.1 mL to each well. Specifically, three types of PVA nanofibers synthesized from the PVA grades: EG-40P, K-440, and T-330, were evaluated. The cells were then incubated for 4 h in a CO_2_ incubator. After another wash with HBSS, a mixture of cell culture medium and WST-8 reagent from the Cell Counting Kit-8 kit (Dojindo Laboratories, Kumamoto, Japan). was added to each well in a ratio of 10:1 (*v/v*). Following incubation for 30 min in the CO_2_ incubator, OD_450_ was measured using a GloMax Multi Detection System microplate reader (Promega).

## 3. Results and Discussion

### 3.1. Characterization of DOTAP/siRNA-Containing PVA Nanofibers

**Figure 1:**
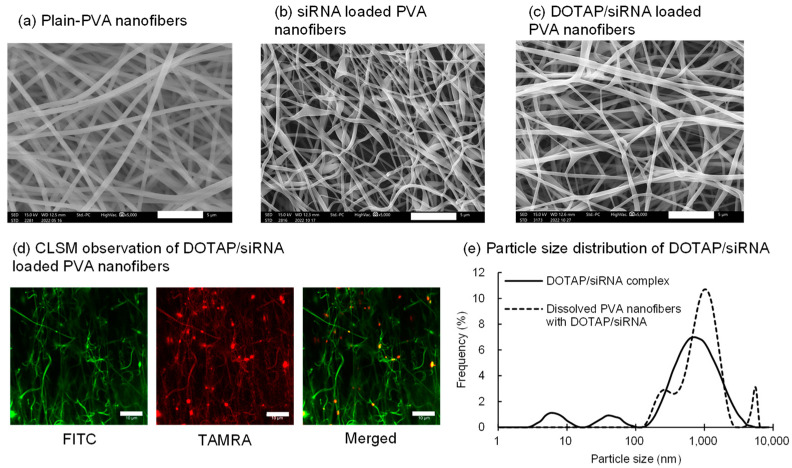
Characteristics of the DOTAP/siRNA-containing PVA nanofibers (N/P ratio = 5) prepared by electrospinning of 8% (*w/w*) PVA (EG-40P) aqueous solution. The nanofibers were prepared using Method A. (**a**) Scanning electron microscope (SEM) images of the PVA nanofibers without encapsulated siRNA (plain PVA nanofibers). (**b**) SEM images of the PVA nanofibers containing naked siRNA (N/P ratio = 0). (**c**) SEM images of the DOTAP/siRNA-containing PVA nanofibers. Scale bar: 5 μm. (**d**) Confocal laser scanning microscope images of the DOTAP/TAMRA-siRNA-containing FITC-PVA nanofibers. Scale bar: 10 μm. (**e**) Comparison of the particle size distribution of the DOTAP/siRNA complexes immediately after preparation and after being released from the PVA nanofibers, which were completely dissolved in water.

Preliminary studies evaluated the particle size and zeta potential of the DOTAP/siRNA complexes prepared by varying the N/P ratio from 0.5 to 10. In general, positively charged nanoparticles interact strongly with the oppositely charged cell membranes and are readily internalized by the cells via endocytosis [29]. At an N/P ratio of 5, the complexes exhibited a high positive charge (30.1 mV), and therefore, they were used. Nanofibers electrospun solely from PVA (EG-40P) solution yielded relatively linear fibers with an average diameter of 473 ± 57 nm (Figure 1a). The siRNA encapsulation efficiency in PVA nanofibers, measured using HPLC, was 94.5 ± 1.5%. This elevated drug encapsulation efficiency in electrospun fibers could be attributed to the drug and polymer solubilities in water, thus minimizing drug loss during encapsulation [31,32]. Incorporation of either siRNA or DOTAP/siRNA complexes (N/P ratio = 5) slightly reduced the fiber diameters to 382 ± 43 nm and 311 ± 88 nm, respectively (Figure 1b,c), presumably because of the enhanced conductivity of the PVA solution [33].

CLSM observations were conducted on fibers containing FITC-PVA and TAMRA-siRNA to observe the distribution of lipoplexes within the nanofibers (Figure 1d). The punctate dispersion of TAMRA-siRNA fluorescence within the PVA nanofibers confirmed the uniform distribution of the DOTAP/siRNA complexes. Subsequently, the particle size distribution and zeta potential of DOTAP/siRNA-containing PVA nanofibers dissolved in Milli-Q water were compared with those of the freshly prepared complexes. This experiment ascertained whether DOTAP/siRNA was released from the PVA nanofibers. Figure 1e shows a comparison of the particle size distribution of the DOTAP/siRNA complexes immediately after preparation and release from PVA nanofibers, which were completely dissolved in water. Due to the simple mixing of each solution, the freshly prepared DOTAP/siRNA complexes exhibited multiple peaks in the submicron range, lacking precise particle size control. Although there was no exact match in the peaks of the freshly prepared DOTAP/siRNA complexes and those released from the dissolved PVA nanofibers, the average particle size for both was <1 μm. This finding suggests the preservation of the integrity of the DOTAP/siRNA complexes within the nanofibers. However, the zeta potential of the freshly prepared complexes was 30.1 mV, while that of the DOTAP/siRNA complexes within the PVA nanofibers was reduced to 4.3 mV. This decline indicates that the residual PVA was adsorbed or remained on the surface of the DOTAP/siRNA complexes released from the nanofibers.

### 3.2. RNAi Efficacy of the DOTAP/siRNA-Containing PVA Nanofibers

**Figure 2:**
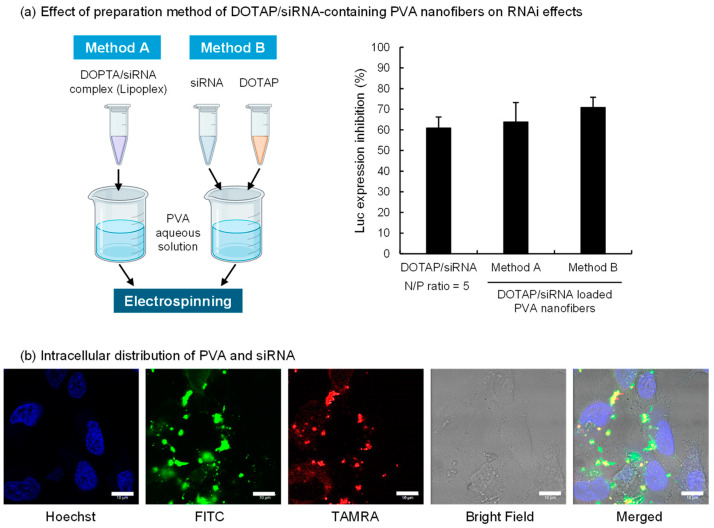
Inhibition of luciferase expression and intracellular distribution of DOTAP/siRNA complexes released from PVA (EG-40P) nanofibers. (**a**) The schematic diagram on the left illustrates the preparation methods of DOTAP/siRNA-containing PVA nanofibers (N/P ratio = 5), created with BioRender.com. The right side of the figure shows the effect of these different preparation methods on the gene expression inhibition rate in A549-Luc cells. The bars in the graph represent mean values ± SD (*n* = 3). (**b**) Intracellular distribution of FITC-PVA and TAMRA-siRNA in A549-Luc cells. CLSM images were taken after 4 h of incubation of cells with the completely dissolved DOTAP/siRNA-containing PVA nanofiber solution using FITC-PVA and TAMRA-siRNA in a serum-free medium. Scale bar: 10 μm.

The RNAi efficacy of DOTAP/Luc-siRNA-containing nanofibers was evaluated based on the suppression of luciferase expression in A549-Luc cells (Figure 2a). As expected, nanofibers containing naked siRNA (N/P ratio = 0) did not show any inhibition. DOTAP/siRNA complexes at an N/P ratio of 5 inhibited luciferase expression by ~60%.

The two preparation methods of the PVA nanofibers tested are represented in Figure 2a. Method A involved mixing the DOTAP/siRNA complex with the PVA solution before spinning, whereas Method B entailed adding the DOTAP suspension and siRNA solution separately into the PVA solution. The morphology of the DOTAP/siRNA-containing PVA nanofibers was not notably affected by the methods (Figure S1). The effect of RNAi on A549-Luc cells was verified using both methods, which revealed no marked variation in the inhibition of luciferase expression. This finding suggests that bypassing the lipoplex preparation process and directly mixing cationic lipids with nucleic acids in a PVA aqueous solution facilitated siRNA delivery into the cells. In Method B, DOTAP and siRNA in the PVA solution could potentially form complexes. This approach simplified the nanofiber fabrication process, which is critical for scaling up production. This study evaluated the impact of siRNA after 24 h of adding the samples to A549-Luc cells. However, the RNAi activity can vary with incubation time. Therefore, future studies should assess the time course of RNAi efficacy for DOTAP/siRNA released from PVA nanofibers.

Subsequently, the intracellular behavior of DOTAP/siRNA-containing PVA nanofibers prepared via Method A on A549-Luc cells was assessed using CLSM (Figure 2b). Method A, which involves preparing the DOTAP/siRNA complexes before adding them to the PVA solution, was adopted to evaluate the intracellular behavior of DOTAP/siRNA complexes released from PVA nanofibers. Considering the possibility that PVA remained attached to the released DOTAP/siRNA complexes, the intracellular distribution of FITC-PVA and TAMRA-siRNA was ascertained. A549-Luc cells were exposed to a serum-free medium containing PVA nanofibers, followed by a 4 h incubation and observation with CLSM. The results indicated siRNA within the cytoplasm of A549-Luc cells, with PVA detected inside the cells, often colocalized with siRNA. This observation suggests that the DOTAP/siRNA complexes with adhered PVA were internalized into the cells via endocytosis. Thus, the grade of PVA, including its degrees of polymerization and hydrolysis, influences the interactions between DOTAP/siRNA complexes and cells [34].

### 3.3. Impact of PVA Grades on the DOTAP/siRNA-Containing PVA Nanofibers

**Figure 3:**
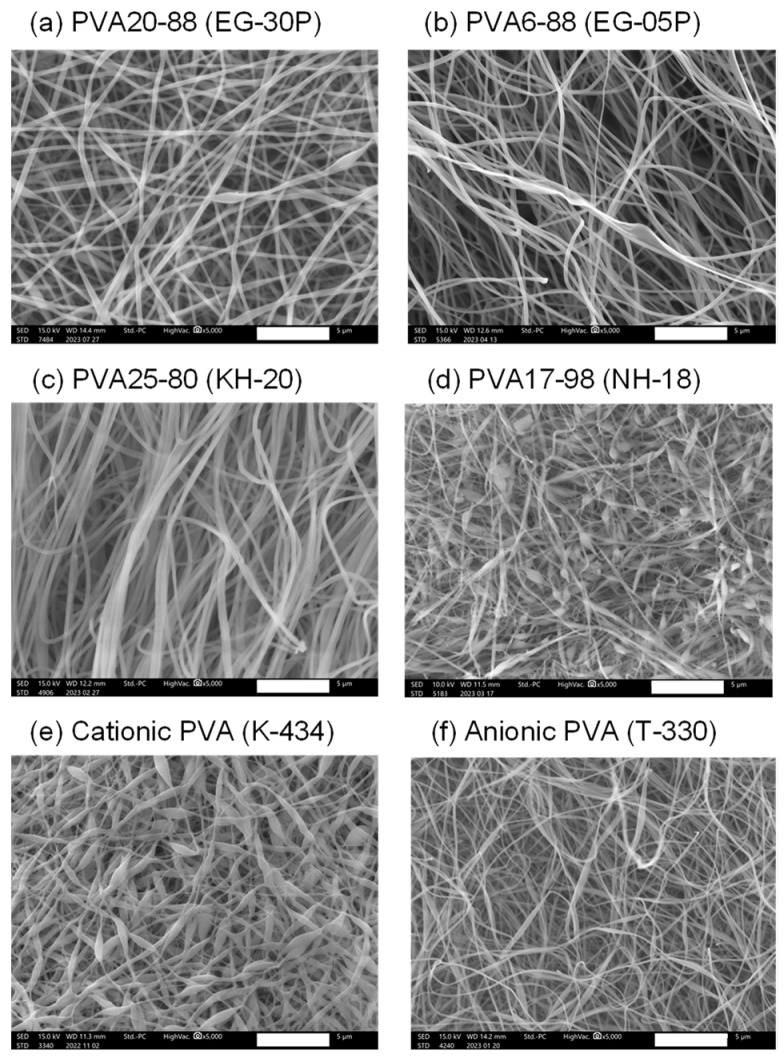
Scanning electron microscope (SEM) images of DOTAP/siRNA-containing PVA nanofibers (N/P ratio = 5) using different grades of PVA. PVA nanofibers were prepared using Method A. Scale bar: 5 μm. (**a**) PVA20-88 (EG-30P), (**b**) PVA6-88 (EG-05P), (**c**) PVA25-80 (KH-20), (**d**) PVA17-98 (NH-18), (**e**) Cationic PVA (K-434), (**f**) Anionic PVA (T-330). Each SEM image corresponds to the nanofibers listed in Table 1.

The nanofibers containing DOTAP/siRNA (N/P ratio = 5) were fabricated by employing the electrospinning method A with PVAs of different degrees of polymerization, saponification, and charges to investigate the impact of various types of PVA on the intracellular delivery of nucleic acids. Table 1 lists the characteristics of the PVAs used in this experiment. As shown in Figure 2a, there was no remarkable difference in RNAi efficacy between Methods A and B. Therefore, Method A was adopted for this study. However, for future investigations, it will be necessary to prepare nanofibers with different PVA grades by employing Method B and evaluating their RNAi efficacy. Figure 3 presents the SEM images of the nanofibers fabricated with various PVAs. With the exception of EG-05P, an 8% solution of PVA was used for electrospinning. Previous studies by our group have shown that the relatively low molecular weight of EG-05P results in a lesser viscosity of 8%, making it unsuitable for spinning [22]. Therefore, a 20% solution was used for EG-05P in this study (Table 1). Nanofibers were spun from all types of PVA and collected as mats from the plate collector. The nanofibers from NH-18 (fully hydrolyzed) and K-434 (cationic PVA) exhibited bead-on-string structures (Figure 3d,e).

**Figure 4:**
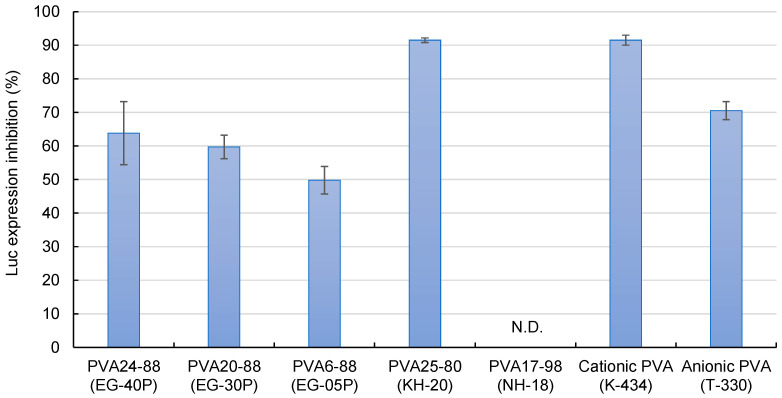
The efficacy of DOTAP/siRNA-containing PVA fibers (N/P ratio = 5) in inhibiting luciferase expression. Different grades of PVA nanofibers were prepared using Method A. Each bar represents the mean value ± SD (*n* = 4).

Figure 4 demonstrates the effects of the DOTAP/siRNA-containing PVA nanofibers on the suppression of luciferase expression in A549-Luc cells. PVA6-88 (EG-05P) demonstrated a lower inhibition rate. Compared to other formulations, with PVA6-88, the higher polymer concentration (20%) in the solution before electrospinning resulted in a greater amount of PVA in the nanofibers. When these PVA6-88 nanofibers were dissolved, more PVA would adhere to the DOTAP/siRNA complexes. This affinity enhanced the presence of PVA on the complexes, which may hinder their interaction with cells, reducing the RNAi activity.

Conversely, the RNAi effect of PVA25-80 (KH-20), a PVA with a low degree of hydrolysis, was 91.5 ± 0.7%, which was higher compared with the partially hydrolyzed PVAs. Low-hydrolysis PVAs contain more acetyl groups, which enhances their hydrophobicity. The elevated presence of hydrophobic PVA on the surface of the DOTAP/siRNA complexes facilitated the cell internalization of the complexes. The fully hydrolyzed PVA17-98 (NH-18) did not inhibit luciferase expression. Fully hydrolyzed PVA has lower water solubility owing to the intermolecular and intramolecular hydrogen bonding among the hydroxyl groups of the PVA molecules being more substantial than their interactions with water, limiting the release of DOTAP/siRNA from the nanofibers [35].

Cationic (K-434) and anionic (T-330) PVAs are mainly used in industrial applications but not as pharmaceutical excipients [36,37,38]. The suppression of luciferase expression was relatively high for these types of PVA, particularly cationic PVAs. The presence of charged PVA near the surface of the DOTAP/siRNA complexes enhances their interaction with the cells. For instance, fabricating nanofibers blended with cationic polymers such as chitosan and PVA can potentially maximize the efficacy of intracellular siRNA delivery [10,29].

**Figure 5:**
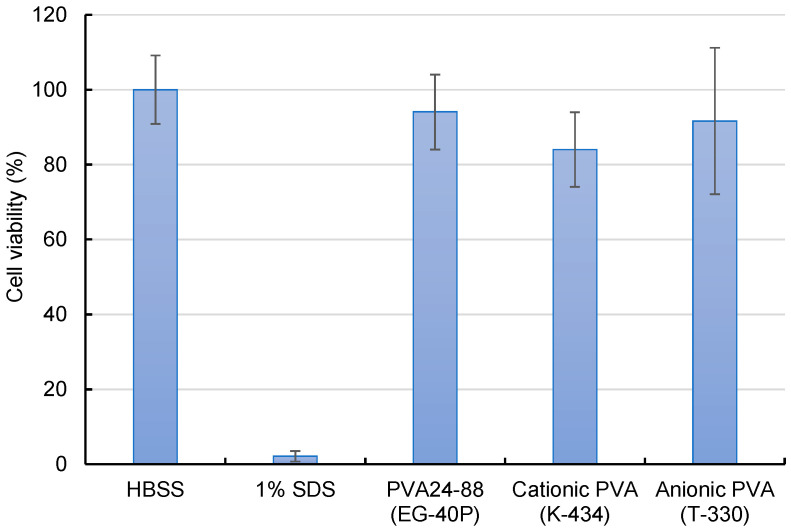
Cytotoxicity evaluation of A549-Luc cells treated with DOTAP/siRNA-containing PVA nanofibers (N/P ratio = 5) by employing the WST-8 assay. A549-Luc cells were exposed to various samples containing 100 nM siRNA for 4 h, and their viability was measured. As a positive control, 1% sodium dodecyl sulfate was also evaluated. Bars in the graph represent the mean values ± SD (*n* = 4–5).

Assessment of the cytotoxicity of cationic and anionic PVA nanofibers revealed a slight decline in the cell viability of cationic PVA (Figure 5). Although cationic PVA allowed efficient intracellular siRNA delivery, the potential for marginal cell damage suggests further consideration.

## 4. Conclusions

This study aimed to develop a nucleic acid drug delivery system using electrospun PVA nanofibers by encapsulating DOTAP/siRNA complexes within the nanofibers and elucidating the characteristics of siRNA-encapsulated nanofibers, including their RNAi activity. It was demonstrated that electrospinning efficiently encapsulated DOTAP/siRNA complexes within the PVA nanofibers. Stable inhibition of luciferase expression was observed, even when the DOTAP/siRNA complex preparation process was bypassed by directly adding each component to the PVA solution. Furthermore, DOTAP/siRNA complexes released from the PVA nanofibers bore PVA residues on their surfaces, suggesting that the properties of PVA may influence interactions between the complexes and cells. Therefore, the degree of hydrolysis and polymerization, or the grade of PVA, affected luciferase expression inhibition, with a higher RNAi activity observed using low hydrolysis degree PVA. Although modified PVA carrying a charge is not currently authorized as a pharmaceutical excipient, the intense inhibition of luciferase expression demonstrated that blending charged polymers with PVA could potentially create more efficient nanofibers for the delivery of nucleic acid drugs to cells.

## Figures and Tables

**Table 1:** Characteristics of different PVA grades and the respective PVA concentrations for electrospinning used in this study.

Characterization of PVA					PVA Concentration in Solution(%) Used for Electrospinning
Abbreviation	PVA Grade	Degree of Polymerization	Degree of Hydrolysis (mol %)	Note	
PVA24-88	GOHSENOL™EG-40P	2400	88		8
PVA20-88	GOHSENOL™EG-30P	2000	88		8
PVA6-88	GOHSENOL™EG-05P	600	88		20
PVA25-80	GOHSENOL™KH-20	2500	80		8
PVA17-98	GOHSENOL™NH-18	1700	98		8
Cationic PVA	GOHSENX™ K-434	1500	88	Modified PVA having a cationic group (quaternary ammonium salt)	8
Anionic PVA	GOHSENX™ T-330	1900	95	Modified PVA having a carboxyl group	8

## Data Availability

Data are contained within the article and Supplementary Materials.

## Supplementary Materials

The following supporting information can be downloaded at: https://www.mdpi.com/article/10.3390/nano14131083/s1, Figure S1: Comparison of SEM images of DOTAP/siRNA-containing PVA nanofibers prepared using different methods (Method A and B) as shown in Figure 2. PVA (EG-40P) was used, and the N/P ratio of DOTAP/siRNA was 5. The SEM image on the left (Method A) corresponds to Figure 1c in the main text.
