# Supplementary material for: Development of Polyvinyl Alcohol (PVA) Nanofibers Containing Cationic Lipid/siRNA Complexes via Electrospinning: The Impact of PVA Characterization
Source: Nanomaterials (Basel). 2024 Jun 24;14(13):1083. doi: 10.3390/nano14131083 (PMC11243518; doi:10.3390/nano14131083)
Supplement: Supplementary file 1 [file nanomaterials-14-01083-s001.zip › nanomaterials-3016332-supplementary.pdf]

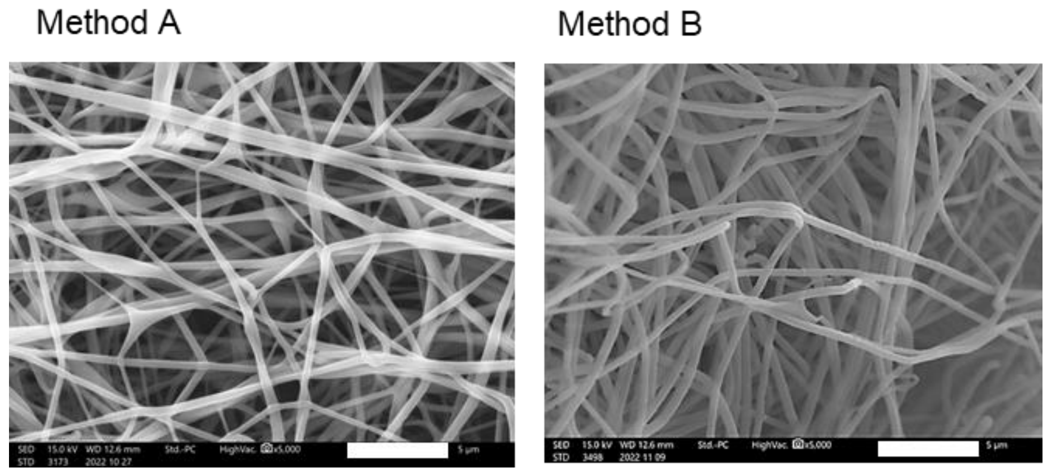

Supplementary Figure S1

Comparison of SEM images of DOTAP/siRNA-containing PVA nanofibers prepared using different methods (Method A and B) as shown in Fig. 2. PVA (EG-40P) was used, and the N/P ratio of DOTAP/siRNA was 5. The SEM image on the left (Method A) corresponds to Figure 1c in the main text.
